# It More than Adds Up: Interaction of Antibiotic Mixing and Temperature

**DOI:** 10.3390/life11121435

**Published:** 2021-12-20

**Authors:** Marie-Claire Danner, Sharon Omonor Azams, Anne Robertson, Daniel Perkins, Volker Behrends, Julia Reiss

**Affiliations:** 1 School of Life and Health Sciences, Whitelands College, University of Roehampton, London SW15 4JD, UK; dannermarieclaire@gmail.com (M.-C.D.); azamss@roehampton.ac.uk (S.O.A.); A.Robertson@roehampton.ac.uk (A.R.); daniel.perkins@roehampton.ac.uk (D.P.); Volker.Behrends@roehampton.ac.uk (V.B.); 2 FRB—CESAB, Institut Bouisson Bertrand, 34070 Montpellier, France

**Keywords:** *Pseudomonas fluorescens*, dose–response, ED_50_, additive models, independent action, concentration addition, antibiotics, temperature

## Abstract

Use of antibiotics for the treatment and prevention of bacterial infections in humans, agri- and aquaculture as well as livestock rearing leads to antibiotic pollution of fresh water and these antibiotics have an impact on free-living bacteria. While we know which antibiotics are most common in natural environments such as rivers and streams, there is considerable uncertainty regarding antibiotics’ interactions with one another and the effect of abiotic factors such as temperature. Here, we used an experimental approach to explore the effects of antibiotic identity, concentration, mixing and water temperature on the growth of *Pseudomonas fluorescens*, a common, ubiquitous bacterium. We exposed *P. fluorescens* to the four antibiotics most commonly found in surface waters (ciprofloxacin, ofloxacin, sulfamethoxazole and sulfapyridine) and investigated antibiotic interactions for single and mixed treatments at different, field-realistic temperatures. We observed an overall dependence of antibiotic potency on temperature, as temperature increased efficacy of ciprofloxacin and ofloxacin with their EC_50_ lowered by >75% with a 10 °C temperature increase. Further, we show that mixtures of ciprofloxacin and ofloxacin, despite both belonging to the fluoroquinolone class, exhibit low-temperature-dependent synergistic effects in inhibiting bacterial growth. These findings highlight the context dependency of antibiotic efficacy. They further suggest antibiotic-specific off-target effects that only affect the bacteria once they enter a certain temperature range. This has important implications as freshwater systems already contain multi-drug antibiotic cocktails and are changing temperature due to environmental warming. These factors will interact and affect aquatic food webs, and hence this creates an urgent need to adapt and improve laboratory testing conditions to closer reflect natural environments.

## 1. Introduction

Freshwater micro-organisms are exposed to ever-increasing levels of antibiotic pollution [1], and some antibiotics have been shown to occur in particularly high concentrations in the environment [2]. Antibiotics such as ciprofloxacin, ofloxacin and sulfamethoxazole are prevalent in European surface waters and frequently measured in concentrations of around 0.01 µg/mL (and in much higher concentrations near wastewater effluents) [2]. Standard approaches to estimating the toxicity/efficacy of antibiotics include constructing dose–response curves for single antibiotics [3], estimating parameters such as minimum inhibitory concentration (MIC) [4] and assessing the half maximal effective concentration (EC_50_) [3]. These methods are important when it comes to exploring the full range of antibiotic toxicity but typically ignore that antibiotics occur as mixtures in polluted environments and that their effects are also density and temperature dependent [2,5].

A complex angle to antibiotic pollution in the environment is that organisms are faced with ‘antibiotic cocktails’; different antibiotics, often of different functional classes, are typically detected simultaneously in fresh water (e.g., [2,6]). The individual concentrations of antibiotics that are measured in the environment might be low, but the combined concentrations could result in significant toxicity and antibiotic resistance as the latter can be the consequence of weak, non-lethal selective pressures such as low levels of antibiotics [7,8]. Chemicals in mixtures potentially interact with each other, which can lead to synergy (the same inhibition is achieved at lower combined concentrations of the mixed antibiotic than for each single antibiotic), additivity (mixed or single effects are identical at the same concentration) or antagonism/suppression (less inhibition is achieved in a mixture than for single antibiotics) [9,10]. It is therefore essential to investigate the potential interactions between antibiotics in the environment [11,12]. It is not straightforward to predict the effects of antibiotic mixtures because the drug effects are dose dependent, non-linear and can affect physiology as well as behavioral traits such as virulence [13]. Therefore, an understanding of the dose–response pattern is required, together with effects of interacting factors such as population densities and temperature [14].

Antibiotics affect different parts of bacterial processes (e.g., protein synthesis or mRNA transcription), which are also influenced by abiotic factors such as pH, nutrient availability or temperature [14]. Despite this, there is limited understanding of how physiological adaptation and stress responses to abiotic stressors affect drug susceptibility in bacteria. While, as a whole, life on Earth can be found across a temperature range of about 150 °C [15], organisms have evolved to grow at their niche-specific ‘optimal’ temperature and changes in growth conditions can be expected to interact with the effects of exogenous stressors such as antibiotics because of organisms’ physiologies [16]. Temperature therefore is a key factor to include in antibiotic studies because both chemical reactions and the metabolic activity of organisms are governed by strict physical laws [16]. In aquatic ecology, theoretical frameworks that include temperature and traits of organisms are established and can explain how communities respond to temperature changes (e.g., [17]). These temperature changes might trigger physiological responses that can be beneficial or detrimental to an organism’s response to antibiotic-induced stress.

As a case in point, Cruz-Loya and colleagues (2019) showed that bacterial response to antibiotic–temperature interaction is complex and mechanism dependent [14]. The response to DNA gyrase-inhibiting ciprofloxacin exposure is linked to the cellular cold shock response (as both cold temperature and gyrase inhibition inhibit unwinding of DNA), while exposure to drugs leading to protein misfolding (usually a feature of higher temperature [15]) is ameliorated by heat shock responses [14]. This context dependency of antibiotic tolerance and its link to temperature is a current area of research, but most studies that address the interaction of antibiotics and temperature (reviewed in [5]) test extreme temperature ranges that are not realistic for free-living bacteria.

Although it is obvious that water temperature, antibiotic type and concentration as well as antibiotic mixture interact in their effects on bacteria, a more mechanistic understanding of these interactions is lacking. Here, we used an experimental approach to explore these drivers on bacterial growth response in *Pseudomonas fluorescens*. First, we determined MICs and concentration-dependent growth inhibition of four antibiotics at higher temperature (25 °C and 30 °C). As a second step, we combined two antibiotics (ciprofloxacin and ofloxacin) in tandem (with a focus on concentrations below the MIC and EC_50_ values, i.e., potentially sub-lethal conditions) at a range of environmental temperatures (from 15 to 25 °C).

## 2. Materials and Methods

### 2.1. Growth and Antibiotic Assays

*Pseudomonas fluorescens* SBW25 [8] was first grown overnight in 75% Luria broth (LB; Sigma-Aldrich; 100% LB contains 10 g/L tryptone; 5 g/L yeast extract, 5 g/L NaCl). This bacterial strain was chosen as it is a free-living organism that occurs in fresh water and is also a widely used test organism regarding pharmaceutical agents [8]. Main cultures in 96 deep-well microplates (2 mL, Corning) were inoculated from overnight to a starting density of 10^5^ cells/mL in 75% LB (except for experiment 2 where densities were 10^6^ cells/mL as this density was more straightforward to adjust from the stock densities). Importantly, these initial concentrations are below stationary phase densities of around 10^9^ cells/mL. *P. fluorescens* was exposed to four antibiotics (and their combinations) chosen because they are the most prevalent in polluted fresh waters [2]. Ciprofloxacin and ofloxacin are both classified as fluoroquinolones which inhibit DNA replication and transcription, sulfamethoxazole inhibits the enzyme dihydropteroate synthetase (DHPS) and sulfapyridine is a folic acid metabolism inhibitor necessary for cell division.

**Figure 1:**
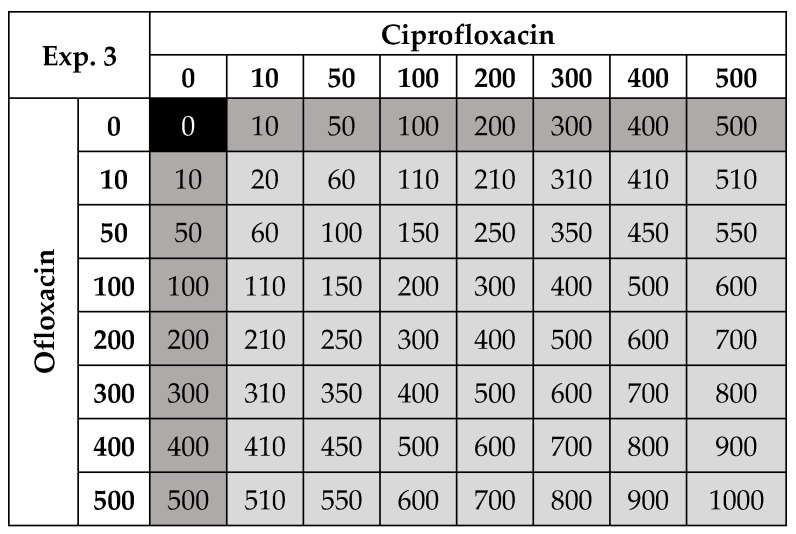
Experimental design used for four temperatures (15–25 °C) where ciprofloxacin and ofloxacin were run as single antibiotic treatments (dark grey boxes) and in combination (light grey boxes) to estimate the effect on *P. fluorescens* densities. All numbers are µg/L and the combined concentrations are shown in the light grey boxes. The control is highlighted in black. There were 64 different antibiotic and concentration combinations (including the bacterial control), replicated 3 times, for four temperatures, resulting in 768 microcosms. This set-up includes 49 antibiotic mixtures where ciprofloxacin and ofloxacin are present in different proportions (33 different proportions).

MICs of ciprofloxacin, ofloxacin, sulfamethoxazole and sulfapyridine, all obtained from Sigma-Aldrich, were determined by measuring growth (optical density at 630nm) in the presence of different antibiotic concentrations (0.01 to 128,000 µg/L) at 30 °C (experiment 1) and 25 °C (experiment 2) after 24 h (Table 1). To assess near field-realistic concentrations and temperature, we grew bacteria in ciprofloxacin and ofloxacin mixtures of up to a 1000 µg/L combined concentration (Table 1) in a fully factorial design (Figure 1) at four temperatures: 15, 17.5, 20 and 25 °C (experiment 3). In the latter experiment, different incubation temperatures were achieved by running four environmental chambers (thermostatic cabinet, Lovibond^®^) set to 15, 17.5, 20 and 25 °C, which are all ecologically relevant as they are within the range of natural variation in fresh waters and are sub-optimal/optimal temperatures for growth. As temperature was hence confounded with a cabinet, we made sure to run replicates in time blocks (i.e., replicates were run on different days) and this enabled us to replicate temperature treatments. The environmental chambers had a light source set to a 12 h dark and 12 h light cycle. For all experiments, growth was assayed after 24 h.

### 2.2. Dose–Response, Antibiotic–Temperature and Antibiotic–Antibiotic Interaction

We constructed dose–response curves for single antibiotics in all experiments following the dose–response method described by Ritz and colleagues [3]. Analysis of the dose–response curves was performed using the drc package for the R statistical environment [18] developed by these authors. The code provided by the authors allows the user to analyze various dose–response models for different types of data. Further, the approach by Ritz et al. (2015) has the advantage that it can output several parameters for summarizing fitted models and carrying out inference on derived parameters such as EC_50_ (half maximal effective concentration), also called ED_50_ (effective dose—this term is used by Ritz et al. [3]). Supplementary Table S1 gives more information about the dose–response method. The significance of the factors ‘temperature’ and ‘antibiotic’ on dose–response parameters was tested by comparing the fit of nested models.

The effects of temperature were explored mainly for experiment 3 across four temperatures by comparing bacterial growth for control and single antibiotics, by contrasting EC_50_ values of single antibiotics and by comparing antibiotic–antibiotic interactions.

For antibiotic–antibiotic interactions, we first performed a synergy check for the 30 °C data (experiment 1) using a serial dilution checkerboard approach. To analyze data from the interaction experiment at near field-realistic concentrations (experiment 3), we chose a simple and visual ‘expected OD’ approach. As the antibiotics used have the same mode of action, additive effects were to be expected and mathematical models exist [18]. Here, we apply, to our knowledge, a novel way to visualize additivity, synergy or antagonism by plotting the integral between the two single antibiotic dose–response curves and checking if the observed potency of mixtures falls within that integral (additivity) and/or exceeds (antagonism) and/or dips below (synergy) expected values. For these ‘expected OD’ plots, all replicates were averaged.

All statistical analyses were performed in R statistical software [19] including the production of figures.

## 3. Results

### 3.1. MIC and Dose–Response (EC_50_)

**Figure 2:**
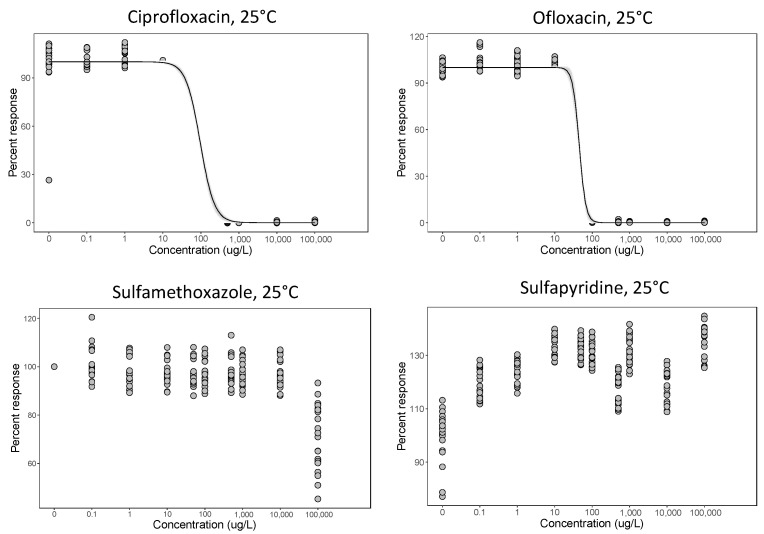
Dose–response curves for a range of concentrations of four single antibiotics at 25 °C (experiment 2). The effect of the concentration on *P. fluorescens* densities was measured as OD and is expressed as a percentage of the control.

Experiment 1 (30 °C) showed that ciprofloxacin and ofloxacin had MICs of under 500 ug/L at this temperature, while sulfamethoxazole and sulfapyridine concentrations inhibiting bacterial growth were over 16 times higher than that (ciprofloxacin, ofloxacin sulfamethoxazole and sulfapyridine had MICs of 500, 125, 32,000 and 8000 ug/L, respectively, Table 2). Hence, in experiment 2 (25 °C), only ciprofloxacin and ofloxacin exhibited classic dose–response curves (Figure 2). EC_50_ values of the latter two antibiotics were under 400 ug/L at 25 °C but there was no obvious pattern or growth inhibition for sulfamethoxazole and sulfapyridine (Figure 2), as expected from the high MIC values estimated in experiment 1.

### 3.2. Temperature Effects

**Figure 3:**
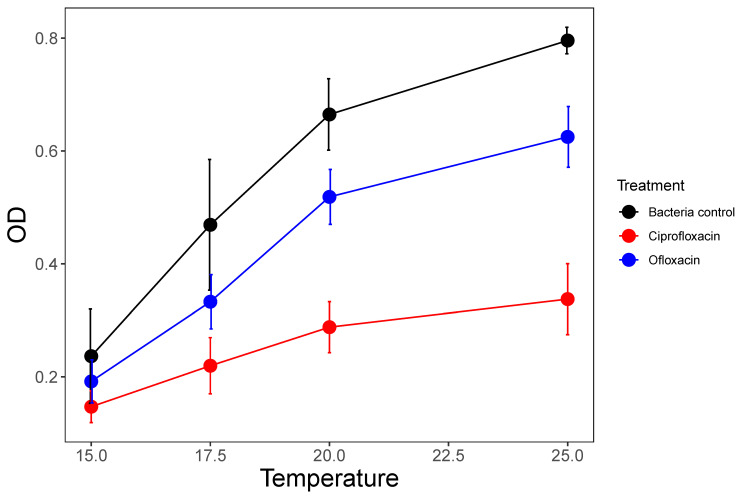
Optical density (means ± SE) at four different temperatures averaged for microcosms with *P. fluorescens* only (control) and those that also contained a single antibiotic. The data shown for the antibiotic treatments are averaged across all concentration treatments from 10 to 500 µg/L.

**Figure 4:**
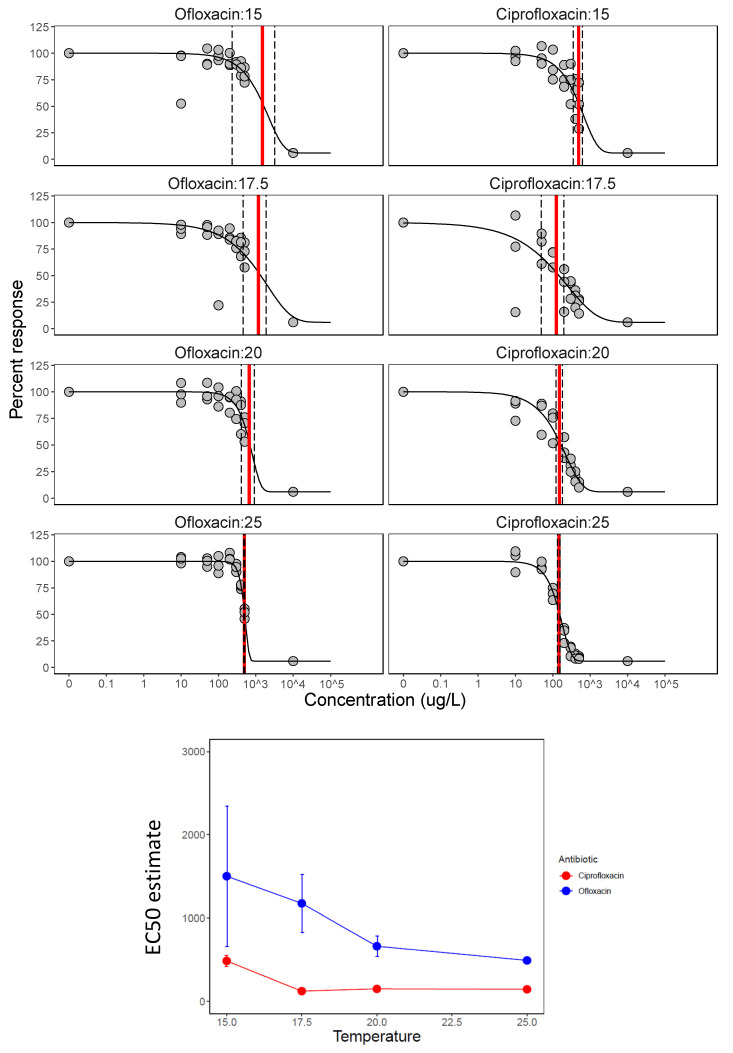
Temperature effects on EC_50_. Upper panels: dose–response curves for a range of concentrations of single antibiotics (ciprofloxacin and ofloxacin) at four temperatures from 15 to 25 °C. The effect of the concentration on *P. fluorescens* densities was measured as OD and is expressed as a percentage of the control. The red line indicates the EC_50_ value with dashed lines showing the confidence intervals, again showing the decrease in EC_50_ with temperature. Lower single panel: EC_50_ decreases with temperature.

Overall, taking all experiments together, temperature increased the potency of antibiotics drastically while also increasing bacterial growth. For example, in experiment 3, bacterial growth responded to temperature in the control and single antibiotic treatments (Figure 3). The shape of the single antibiotic dose–response curves and EC_50_ values were an appropriate tool to compare temperature effects (dose–response essentially corrects for higher growth at higher temperature). Comparing EC_50_ across the four temperatures in experiment 3 (Figure 4) showed that EC_50_ was lowered by >75% with a 10 °C temperature increase, and this was true for both ciprofloxacin and ofloxacin (Table 3, Figure 4). EC_50_ was reached at around 145 and 490 µg/L for ciprofloxacin and ofloxacin, respectively, at 25 °C in this experiment (Table 3). EC_50_ values were even lower in experiment 2 (that also had a higher starting density of bacteria, Table 1, Figure 2) which points to bacteriocidal as well as growth inhibitory effects. Testing for the significance of these findings, there was strong evidence for the effects of antibiotic type and temperature on dose–response curves from experiment 3 (Table S3). Model comparison revealed that the best model included dose–response parameters for each combination of temperature and antibiotic levels (Table S3; Table 2; Figure 4).

### 3.3. Antibiotic Mixing and Temperature

**Figure 5:**
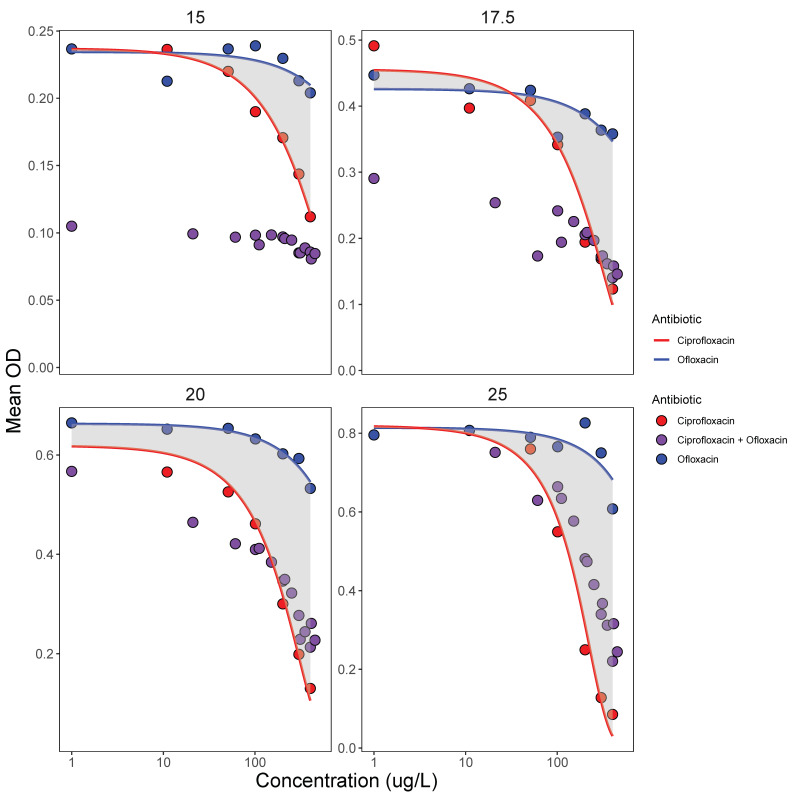
Potency of antibiotic mixtures of ciprofloxacin and ofloxacin (in 33 different proportions) compared to the dose–response of the single antibiotics (importantly, concentration range shown includes concentrations below MIC and EC_50_) for four temperatures. If mixtures (in purple) behave in synergy, bacterial growth will be below the integral of the single antibiotic effects, and this is largely the case for the 15 °C and 17.5 °C treatments (upper two panels). If mixtures behave in an additive fashion, bacterial growth will be within the integral of the single antibiotic effects, and this is largely the case for the 20 °C and 25 °C treatments (lower two panels). All values are means calculated from 3 replicates.

Experiment 1 pointed towards synergy between ciprofloxacin and ofloxacin on the ‘edges’ of the organisms’ ‘thermal breadth’ (Table 2, Supplementary Figure S1) that was then explored in experiment 3. Combining ciprofloxacin and ofloxacin in the latter experiment, antibiotic mixtures showed the same potency as their constituent parts (Figure 5). For example, at 25 °C, bacterial densities, responding to combined concentrations of the mixtures, fall within the integral between the dose–response curves of ciprofloxacin and ofloxacin (Figure 5) and effects are hence largely additive. However, at the two lower temperatures (15 °C and 17.5 °C), synergetic effects are apparent, where bacterial densities were much lower than expected from running ciprofloxacin and ofloxacin in isolation (Figure 5).

Interestingly, this was especially true for the range of concentrations well below the MIC and EC_50_ of the single antibiotics (Figure 5), i.e., sub-lethal concentrations ‘add up’ to inhibit growth. For instance, at 15 °C, combining 10 µg/L of ciprofloxacin and ofloxacin, respectively (i.e., combined concentration of 20 µg/L), results in the same effect as 400 µg/L of ofloxacin on its own.

## 4. Discussion

In this study, we investigated interactions between temperature and antibiotics at sub-MIC concentrations for *P. fluorescens* and showed that this interaction was twofold: at low and very high temperatures (possibly outside the organisms’ temperature optimum), the antibiotic mixtures showed increased synergy, yet overall temperature increased antibiotic efficacy for single antibiotics. A striking result of our study was that antibiotic mixtures had lethal effects even when the concentrations added together were below their respective individual toxicity in a realistic antibiotic pollution scenario.

The latter result was unexpected because ciprofloxacin and ofloxacin both belong to the fluoroquinolone class and are not expected to act in synergy. Antibiotics with different target action are more likely to show interactions when combined—either as synergy [20] or as antagonism/suppression [10]—and there is now growing evidence that both synergy and antagonism are a common feature of antibiotics, and generally pharmaceuticals and other stressors, in mixtures [10,18,21]. Focusing on antibiotic mixing is important as risk assessments and ecotoxicological tests are based on single compounds but antibiotics, in common with all pharmaceuticals, do not occur as isolated and pure substances in the environment and they should be regarded as a multi-component chemical mixture [2]. A growing body of literature shows that mixtures of pollutants can have different effects compared to single compounds and that the joint effect of such chemical cocktails is often higher than the toxicity of each individual compound [12,22,23]. For example, González-Pleiter et al. (2013) demonstrated that the combined effect can be ‘more than the sum of the parts’ by testing the effects of antibiotics in mixtures including a mixture of erythromycin and tetracycline that had particular strong synergistic effects on cyanobacteria [11]. However, knowledge about the toxicity of antibiotic mixtures is still limited and ignoring possible mixture effects might underestimate the actual impact of antibiotics in the environment [20].

We observed low- and high-temperature-dependent synergistic effects in inhibiting bacterial growth, suggesting antibiotic-specific off-target effects that only affect the bacteria once they enter a certain temperature range. This is in line with emerging literature on antibiotic mixing and temperature [5,14,23] that highlights the context dependency of antibiotic efficacy and it alludes to the fact that stressors such as chemicals, temperature or pH interact [5]—especially when levels are reached that are outside or ‘on the edges’ of the organism’s tolerance breadth [14,24]. Complicating matters, these interactions ‘play out’ on different levels of biological organization—from subcellular to the individual and population level. For example, while it is intuitive that low concentrations of antibiotics change populations because they can provoke resistance in bacteria [8], even more complex mechanisms are at play here and antibiotic stress below lethal levels can result in bacterial strains with narrowed temperature breadth and shifted temperature optima [24]—resulting in individuals/populations that are more susceptible to stressors.

In our study, EC_50_ values of single antibiotics decreased with temperature and explanations for this pattern include that both uptake [25] and metabolism [16] of antibiotics increase with temperature. Additionally, high temperatures enhance the toxicity of contaminants (yet, at the same time, enhance the rates of chemical degradation [26]). Further, synergistic effects are possible such as both temperature and (some) antibiotics influencing protein folding and synthesis [27,28]. Cruz-Loya and colleagues (2019) found that cellular responses to temperature stress have likely been evolutionarily co-opted to also respond to many classes of antibiotic stress [14]. A further factor is that a population-level effect could come into play in a nutrient-limited environment, as a rise in temperature results in increased population density and potentially competition for resources. In our experiments, the assays with higher population density resulted in lower EC_50_ values of ciprofloxacin and ofloxacin compared to the experiment with lower bacterial densities. Density-dependent effects in bacteria could therefore be explored more when it comes to antibiotic assays and studies, as is indeed the case for other driving forces of evolution in bacteria such as time, space or disturbance (but see, e.g., [29]).

Interactions among different stressors [21] are at the core of unexpected ecological impact because interactions can lessen or amplify the direct signal effect of each stressor [30]. In this vein, adaptation to both temperature and antibiotics is another future research avenue and a strong focus is needed on sub-lethal antibiotic concentrations as highlighted above. Changes in environmental temperature ‘hit’ multicellular organisms in ‘acute’ ways (such as species extinctions or range shifts [31]) but also shape microbial communities despite their seemingly immediate capability to adapt. For instance, bacterial strains adapted to high temperatures can be more sensitive to certain antibiotics [14] and generally temperature can alter the average body size of microbes (e.g., [32]) and this in turn affects metabolic rates [16,33,34].

## 5. Conclusions

We found that temperature increased the efficacy of ciprofloxacin and ofloxacin and, further, our results point to low- and high-temperature-dependent synergistic effects in inhibiting bacterial growth. To date, fundamental ecological questions regarding the effects of antibiotics and temperature on freshwater communities remain unanswered, and a general assessment of their contribution to community and ecosystem functioning is also required. In particular, we need to know how antibiotic mixtures and temperature affect bacterial growth and adaptation and in turn the food web that is fueled by bacterial production.

## Figures and Tables

**Table 1:** Overview of three experiments used in this study.

Experiment	Antibiotic	Temp.	Antibiotic Concentration	Antibiotic Mixtures	Analysis
**1** *P. fluorescens* 10^5^ cells/mL initial%	Ciprofloxacin, ofloxacin, sulfamethoxazole and sulfapyridine	30	Nine concentrations: 125 to 128,000 µg/L for single antibiotics and the mixtures were run from 1/32× to 4× MIC	Antibiotics on their own and all possible two-way combinations	MIC, checkerboard for antibiotic–antibiotic interaction
**2** *P. fluorescens* 10^6^ cells/mL initial%	Ciprofloxacin, ofloxacin, sulfamethoxazole and sulfapyridine	25	Eleven concentrations: 0.1 to 100,000 µg/L	Antibiotics on their own only	Dose–response and EC_50_
**3** *P. fluorescens* 10^5^ cells/mL initial%	Ciprofloxacin and ofloxacin	15, 17.5, 20, 25	Control and nine ‘field-realistic’ concentrations: 10 to 500 µg/L	Antibiotics on their own and all possible two-way combinations of ciprofloxacin and ofloxacin	Dose–response for C and O, temperature effects on growth and antibiotic–antibiotic interactions

**Table 2:** MIC values (ug/L) for four single antibiotics at 30 °C (experiment 1) and effects in terms of MIC in mixtures where S = synergy and I = independence (antagonism was not observed). S was observed for many of the mixtures below the respective MICs of ciprofloxacin and ofloxacin (e.g., for 125 + 15 µg/L, respectively).

	MIC µg/L	Effect in Mixture			
	Alone				
		Ciprofloxacin	Ofloxacin	Sulfamethoxazole	Sulfapyridine
**Ciprofloxacin**	500	na	S	I	I
**Ofloxacin**	125	S	na	I	I
**Sulfamethoxazole**	32,000	I	I	na	I
**Sulfapyridine**	8000	I	I	I	na

**Table 3:** EC_50_ values (µg/L) estimated from dose–response analysis for ciprofloxacin and ofloxacin for four temperatures (experiment 3), along with the lower and upper bounds of 95% confidence intervals.

Treatment	Estimate_EC50	Std. Error	Lower	Upper
Ciprofloxacin: 15 °C	486	65	352	621
Ciprofloxacin: 17.5 °C	123	36	49	197
Ciprofloxacin: 20 °C	150	14	121	180
Ciprofloxacin: 25 °C	145	6	133	156
Ofloxacin: 15 °C	1502	843	233	3237
Ofloxacin: 17.5 °C	1176	348	459	1893
Ofloxacin: 20 °C	663	124	407	919
Ofloxacin: 25 °C	492	7	477	506

## Data Availability

The raw data and R code generated in the study are available upon request from the corresponding author.

## Supplementary Materials

The following are available online at https://www.mdpi.com/2075-1729/11/12/1435/s1, Figure S1: Checkerboard visualization of a minimum inhibitory concentration (MIC) experiment at 30 °C showing the strong interaction of ciprofloxacin and ofloxacin; Table S1: EC_50_ estimate for C and O, run at 25 °C, *Pseudomonas fluorescens*, 24 h; Table S2: All models for dose–response curves for experiment 3, Table S3. Analysis of dose-response curves from experiment 3.
