# Correction: Danner et al. It More than Adds Up: Interaction of Antibiotic Mixing and Temperature. *Life* 2021, *11*, 1435

**DOI:** 10.3390/life12050695

**Published:** 2022-05-07

**Authors:** Marie-Claire Danner, Sharon Omonor Azams, Anne Robertson, Daniel Perkins, Volker Behrends, Julia Reiss

**Affiliations:** 1 School of Life and Health Sciences, Whitelands College, University of Roehampton, London SW15 4JD, UK; dannermarieclaire@gmail.com (M.-C.D.); azamss@roehampton.ac.uk (S.O.A.); A.Robertson@roehampton.ac.uk (A.R.); daniel.perkins@roehampton.ac.uk (D.P.); Volker.Behrends@roehampton.ac.uk (V.B.); 2 FRB—CESAB, Institut Bouisson Bertrand, 34070 Montpellier, France

The Authors wish to make the following corrections to this paper [1]: 

In the original article, there were mistakes in Figure 1, Figure 3 and Figure 5.

Figure 1: picture was not clear enough

Figure 3: layering was hiding symbols and the legend

Figure 5: layering was hiding the data points

The mistakes were caused during copy-editing. The caption in Figure 1 “are shown as numbers in cursive print” should be replaced with “are shown in the light grey boxes”. The corrected Figures appear below.

**Figure 1:**
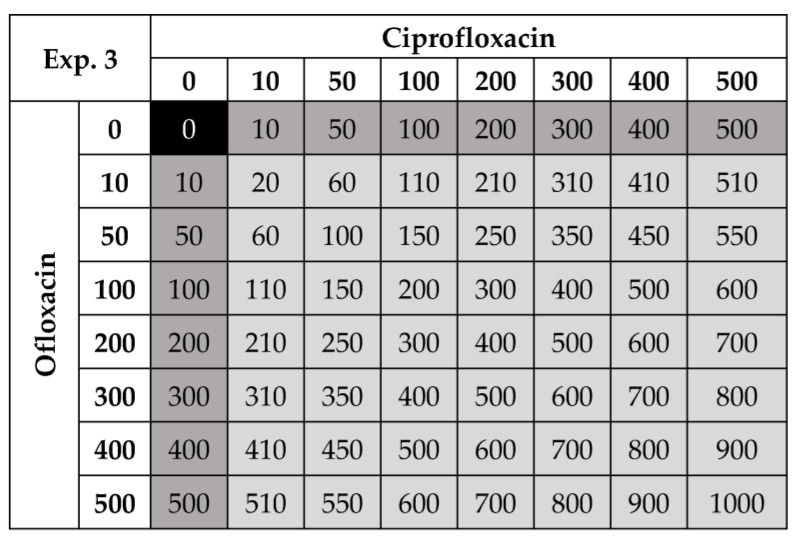
Experimental design used for four temperatures (15–25 °C) where ciprofloxacin and ofloxacin were run as single antibiotic treatments (dark grey boxes) and in combination (light grey boxes) to estimate the effect on *P. fluorescens* densities. All numbers are μg/L and the combined concentrations are shown in the light grey boxes. The control is highlighted in black. There were 64 different antibiotic and concentration combinations (including the bacterial control), replicated 3 times, for four temperatures, resulting in 768 microcosms. This set-up includes 49 antibiotic mixtures where ciprofloxacin and ofloxacin are present in different proportions (33 different proportions).

**Figure 3:**
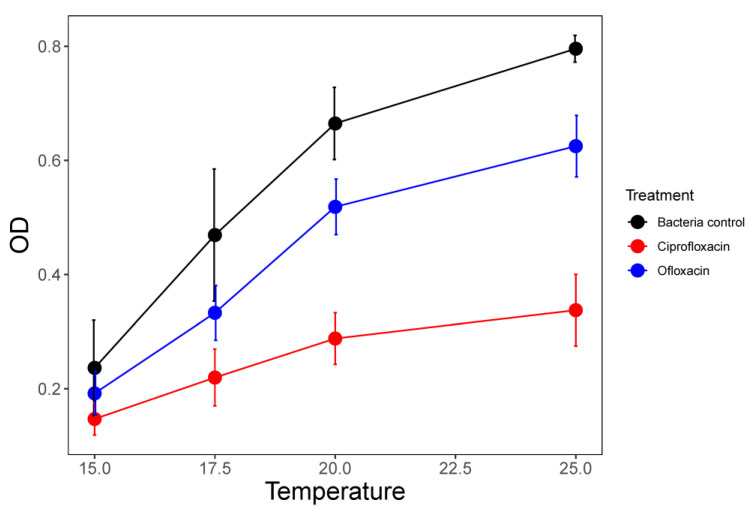
Optical density (means ± SE) at four different temperatures averaged for microcosms with *P. fluorescens* only (control) and those that also contained a single antibiotic. The data shown for the antibiotic treatments are averaged across all concentration treatments from 10 to 500 μg/L.

**Figure 5:**
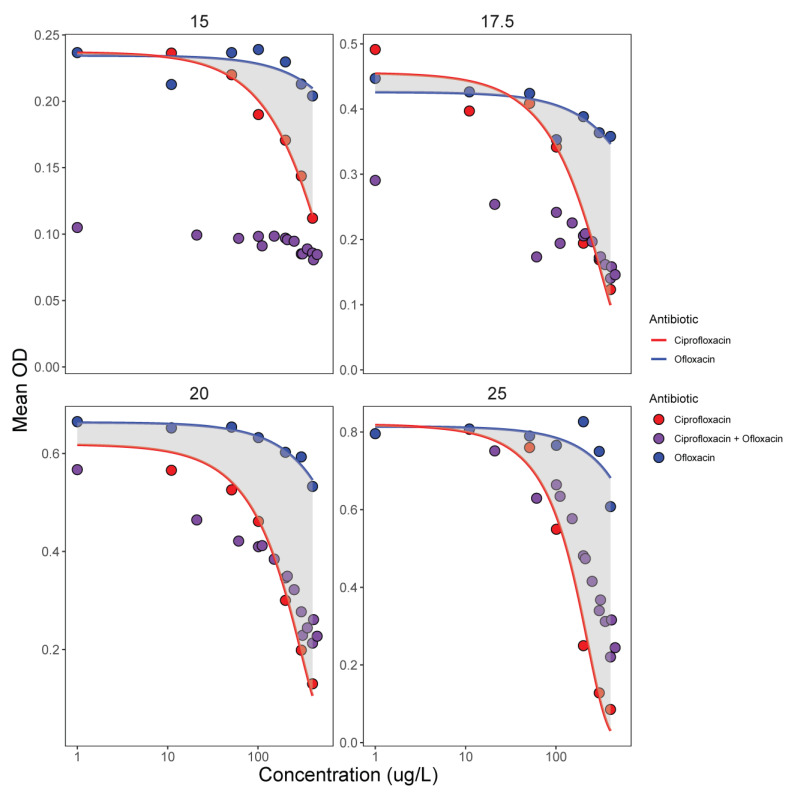
Potency of antibiotic mixtures of ciprofloxacin and ofloxacin (in 33 different proportions) compared to the dose–response of the single antibiotics (importantly, concentration range shown includes concentrations below MIC and EC50) for four temperatures. If mixtures (in purple) behave in synergy, bacterial growth will be below the integral of the single antibiotic effects, and this is largely the case for the 15 °C and 17.5 °C treatments (upper two panels). If mixtures behave in an additive fashion, bacterial growth will be within the integral of the single antibiotic effects, and this is largely the case for the 20 °C and 25 °C treatments (lower two panels). All values are means calculated from 3 replicates.
