# Supplementary material for: It More than Adds Up: Interaction of Antibiotic Mixing and Temperature
Source: Life (Basel). 2021 Dec 20;11(12):1435. doi: 10.3390/life11121435 (PMC8703992; doi:10.3390/life11121435)
Supplement: Supplementary file 1 [file life-11-01435-s001.zip › life-1498518-supplementary.pdf]

Supplementary Information

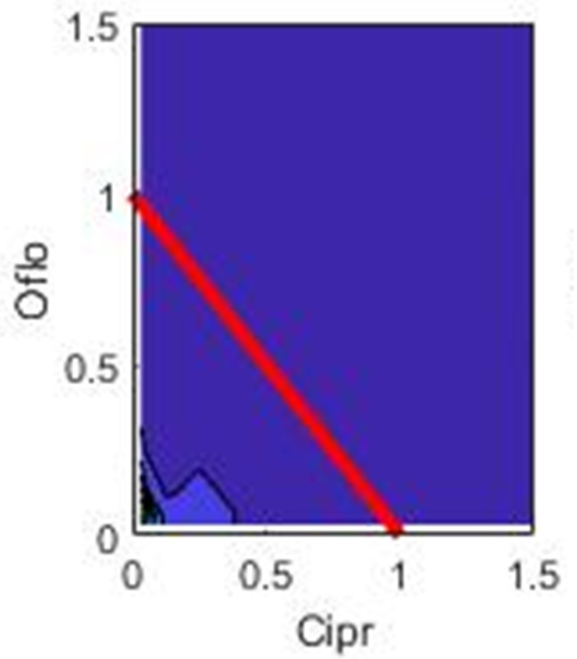

Figure S1: Checkerboard visualisation of a minimum-inhibitory concentration (MIC) experiment at 30°C showing the strong interaction of ciprofloxacin and ofloxacin. The heatmap indicates growth of *P. fluorescence* in treatments that had received a mixture of two antibiotics. A value of 1 indicates the MIC concentration of that particular antibiotic, set to 100%, hence a value of 0.5 indicates 50% of the MIC value. Yellow indicates visible growth of bacteria and cooler colours less growth; and purple indicates no growth. The area to the left-hand side of the 1:1 line (in red) represents mixing where growth is possible in theory if the antibiotics behave in an additive fashion. Combining ciprofloxacin and ofloxacin resulted in very reduced growth in all wells and growth is below what is expected from individual MIC values and proportions in mixture.

Table S1: EC_{50} estimate for ciprofloxacin and ofloxacin, run at 25°C in *Pseudomonas fluorescens*, for 24 hours. We followed the dose-response method described by Ritz and colleagues (Ritz et al., 2015). These authors developed the extension package drc for the statistical environment R (R Development Core Team, 2017). In drc estimation of parameters is based on the maximum likelihood principle, which under the assumption of normally distributed response values simplifies to nonlinear least squares. The codes provided by the authors allow specifying the model assumptions about the dose-response relationship (dose-response models encompass a range of statistical models from nonlinear regression, generalized (non)linear regression, and parametric survival analysis). Further the approach by Ritz et al. (2015) has the advantage that it can output a number of extractors for summarizing fitted models and carrying out inference on derived parameters such as EC_{50} (half maximal effective concentration) or ED50 (effective doses - this term is used by Ritz et al. [2015]). The denotation ED50 is used for continuous responses and this applies in our case but EC_{50} is the common term used in the 'bacteria literature'. Because these models can only be constructed when concentrations are run that result in a plateau of inhibition, we added a dummy data point for all dose-response curves in the main experiment, extrapolating results from the one shown here - that had clearly shown that a plateau was fully reached at concentrations of 10 μg/mL.

**Ofloxacin**					
**Parameter estimates:**	**Estimate**	**Std. Error**	**t-value**	**p-value**	
Slope:(Intercept)	1.817387	0.054604	33.283	< 0.0001	***
Lower Limit:(Intercept)	6.158502	0.27998	21.996	< 0.0001	***
ED50:(Intercept)	112.366533	1.296654	86.659	< 0.0001	***
**Ciprofloxacin**					
**Parameter estimates:**	**Estimate**	**Std. Error**	**t-value**	**p-value**	
Slope:(Intercept)	2.002862	0.077143	25.963	< 0.0001	***
Lower Limit:(Intercept)	6.002552	0.258551	23.216	< 0.0001	***
ED50:(Intercept)	48.613358	0.40359	120.452	< 0.0001	***

Table S2: All models for dose-response curves for the experiment with four temperatures. These are dose-response models for the single antibiotics and the 50:50 mixture.

Treatment	Model	logLik	IC	Lack of fit	Res var
Ciprofloxacin:15	Log-logistic (3 parameter)	-103.55301	213.106024	0.99693745	135.595672
Ciprofloxacin:20	Log-logistic (3 parameter)	-98.757085	203.514171	0.26928477	95.0519539
Ciprofloxacin:25	Log-logistic (3 parameter)	-82.209292	170.418583	0.41720594	27.9009611
Ciprofloxacin + Ofloxacin:15	Log-logistic (3 parameter)	-93.088149	192.176299	0.69479072	62.4585065
Ciprofloxacin + Ofloxacin:17.5	Log-logistic (3 parameter)	-88.952808	183.905616	0.1623297	45.9788206
Ciprofloxacin + Ofloxacin:20	Log-logistic (3 parameter)	-94.835672	195.671343	0.04074909	71.0901318
Ciprofloxacin + Ofloxacin:25	Log-logistic (3 parameter)	-80.880459	167.760919	0.26537109	25.2854495
Ofloxacin:15	Log-logistic (3 parameter)	-101.0086	208.017207	0.27495962	112.303245
Ofloxacin:17.5	Log-logistic (3 parameter)	-110.59723	227.194468	0.30594334	228.485255
Ofloxacin:20	Log-logistic (3 parameter)	-96.636677	199.273354	0.95585323	81.2358253
Ofloxacin:25	Log-logistic (3 parameter)	-78.033656	162.067312	0.11720093	20.4780749
Ciprofloxacin:17.5	Log-logistic (3 parameter)	-115.62394	237.247878	0.53372713	331.563788
Ciprofloxacin:15	Log-logistic (4 parameter)	-103.45943	212.91887	0.99829418	134.659021
Ciprofloxacin:17.5	Log-logistic (4 parameter)	-115.85912	237.71824	0.49333324	337.390493
Ciprofloxacin:20	Log-logistic (4 parameter)	-98.982678	203.965356	0.24470915	96.6536718
Ciprofloxacin:25	Log-logistic (4 parameter)	-78.22245	162.444901	0.99283589	20.7664679
Ciprofloxacin + Ofloxacin:15	Log-logistic (4 parameter)	-92.759535	191.51907	0.75415588	60.9565087
Ciprofloxacin + Ofloxacin:17.5	Log-logistic (4 parameter)	-89.042876	184.085752	0.15571737	46.2866032
Ciprofloxacin + Ofloxacin:20	Log-logistic (4 parameter)	-95.675481	197.350961	0.02603795	75.6529633
Ciprofloxacin + Ofloxacin:25	Log-logistic (4 parameter)	-79.012603	164.025207	0.53700043	22.0182007
Ofloxacin:15	Log-logistic (4 parameter)	-101.15323	208.306451	0.25873125	113.512787
Ofloxacin:17.5	Log-logistic (4 parameter)	-111.06019	228.120385	0.25228752	236.456648
Ofloxacin:20	Log-logistic (4 parameter)	-96.13508	198.27016	0.9880826	78.2728667
Ofloxacin:25	Log-logistic (4 parameter)	-74.755449	155.510899	0.46983	16.0630992
Ciprofloxacin:20	Weibull I (3 parameter)	-96.839451	199.678903	0.55189244	82.4652234
Ciprofloxacin:25	Weibull I (3 parameter)	-88.268137	182.536274	0.02364205	43.7050871
Ciprofloxacin + Ofloxacin:15	Weibull I (3 parameter)	-93.760399	193.520798	0.57218144	65.6474471
Ciprofloxacin + Ofloxacin:17.5	Weibull I (3 parameter)	-87.656443	181.312886	0.28647265	41.768974
Ciprofloxacin + Ofloxacin:20	Weibull I (3 parameter)	-91.660777	189.321554	0.19374693	56.1918175
Ciprofloxacin:15	Weibull I (3 parameter)	-103.76516	213.53032	0.99166479	137.743346
Ciprofloxacin + Ofloxacin:25	Weibull I (3 parameter)	-84.006624	174.013247	0.06083321	31.8741979
Ofloxacin:15	Weibull I (3 parameter)	-100.87827	207.756534	0.2902434	111.224222
Ofloxacin:17.5	Weibull I (3 parameter)	-109.56547	225.130944	0.45392859	211.673469
Ofloxacin:20	Weibull I (3 parameter)	-96.737079	199.474158	0.94656129	81.8422423
Ofloxacin:25	Weibull I (3 parameter)	-78.491599	162.983197	0.09380644	21.1846415
Ciprofloxacin:17.5	Weibull I (3 parameter)	-115.18689	236.373785	0.61190359	321.001691
Ciprofloxacin:15	Weibull I (4 parameter)	-103.34187	212.68373	0.99933842	133.491385
Ciprofloxacin:20	Weibull I (4 parameter)	-96.544653	199.089306	0.60482026	80.6839579
Ciprofloxacin:25	Weibull I (4 parameter)	-81.986628	169.973256	0.45227172	27.4445479
Ciprofloxacin + Ofloxacin:15	Weibull I (4 parameter)	-92.820228	191.640456	0.74333584	61.2311738
Ciprofloxacin + Ofloxacin:17.5	Weibull I (4 parameter)	-86.455871	178.911741	0.45530761	38.2147869
Ciprofloxacin + Ofloxacin:20	Weibull I (4 parameter)	-91.391997	188.783994	0.21825177	55.0841232
Ciprofloxacin + Ofloxacin:25	Weibull I (4 parameter)	-77.391941	160.783883	0.82610216	19.5274346
Ofloxacin:15	Weibull I (4 parameter)	-100.92422	207.84843	0.28478361	111.603428
Ofloxacin:17.5	Weibull I (4 parameter)	-109.81349	225.626984	0.41489088	215.598247
Ofloxacin:20	Weibull I (4 parameter)	-96.014096	198.028192	0.99243929	77.5745355
Ofloxacin:25	Weibull I (4 parameter)	-75.345674	156.691349	0.3784651	16.7809622
Ciprofloxacin:17.5	Weibull I (4 parameter)	-115.10806	236.216118	0.62629159	319.132658
Ciprofloxacin:17.5	Weibull 2 (3 parameter)	-116.69593	239.391856	0.36342213	358.965688
Ciprofloxacin:20	Weibull 2 (3 parameter)	-101.19045	208.380905	0.0877469	113.826238
Ciprofloxacin:25	Weibull 2 (3 parameter)	-80.768313	167.536626	0.66632932	25.0762696
Ciprofloxacin + Ofloxacin:15	Weibull 2 (3 parameter)	-93.126665	192.25333	0.68774284	62.6369557
Ciprofloxacin + Ofloxacin:17.5	Weibull 2 (3 parameter)	-92.01645	190.032901	0.03517391	57.6919324
Ciprofloxacin + Ofloxacin:20	Weibull 2 (3 parameter)	-98.649614	203.299228	0.00492552	94.2982654
Ciprofloxacin + Ofloxacin:25	Weibull 2 (3 parameter)	-83.840104	173.680209	0.0662205	31.4834522
Ciprofloxacin:15	Weibull 2 (3 parameter)	-103.78072	213.561444	0.99114179	137.902217
Ofloxacin:15	Weibull 2 (3 parameter)	-109.53644	225.072878	0.00322304	211.218738
Ofloxacin:17.5	Weibull 2 (3 parameter)	-112.6745	231.349004	0.12129438	266.491776
Ofloxacin:20	Weibull 2 (3 parameter)	-113.86549	233.730975	0.00024603	291.070215
Ofloxacin:25	Weibull 2 (3 parameter)	-117.41516	240.830326	2.52E-12	378.608788
Ciprofloxacin:17.5	Weibull 2 (4 parameter)	-117.07972	240.159438	0.31218639	369.317143
Ciprofloxacin:20	Weibull 2 (4 parameter)	-101.52037	209.040748	0.07442636	116.642266
Ciprofloxacin:25	Weibull 2 (4 parameter)	-79.360041	164.720082	0.90051271	22.5922188
Ciprofloxacin + Ofloxacin:15	Weibull 2 (4 parameter)	-93.585694	193.171388	0.60370588	64.8033703
Ciprofloxacin + Ofloxacin:17.5	Weibull 2 (4 parameter)	-92.606914	191.213829	0.02563322	60.2712625
Ciprofloxacin + Ofloxacin:20	Weibull 2 (4 parameter)	-99.267827	204.535654	0.00344192	98.7169169
Ciprofloxacin + Ofloxacin:25	Weibull 2 (4 parameter)	-84.918345	175.83669	0.03785853	34.1011717
Ofloxacin:15	Weibull 2 (4 parameter)	-109.25747	224.514932	0.00379158	206.898765
Ciprofloxacin:15	Weibull 2 (4 parameter)	-104.19913	214.398264	0.96902466	142.24319
Ofloxacin:17.5	Weibull 2 (4 parameter)	-113.18799	232.375976	0.09455124	276.823295
Ofloxacin:20	Weibull 2 (4 parameter)	-113.66362	233.327243	0.00027839	286.750215
Ofloxacin:25	Weibull 2 (4 parameter)	-117.26024	240.520479	2.79E-12	374.288788
